# Prevalence of signs of trachoma, ocular *Chlamydia trachomatis* infection and antibodies to Pgp3 in residents of Kiritimati Island, Kiribati

**DOI:** 10.1371/journal.pntd.0005863

**Published:** 2017-09-12

**Authors:** Anaseini Cama, Andreas Müller, Raebwebwe Taoaba, Robert M. R. Butcher, Iakoba Itibita, Stephanie J. Migchelsen, Tokoriri Kiauea, Harry Pickering, Rebecca Willis, Chrissy h. Roberts, Ana Bakhtiari, Richard T. Le Mesurier, Neal D. E. Alexander, Diana L. Martin, Rabebe Tekeraoi, Anthony W. Solomon, for the Global Trachoma Mapping Project

**Affiliations:** 1 International Agency for the Prevention of Blindness, Western Pacific Region, Suva, Fiji; 2 The Fred Hollows Foundation, Sydney, Australia; 3 Centre for Eye Research, University of Melbourne, Melbourne, Australia; 4 Eye Department, Ministry of Health and Medical Services, South Tarawa, Kiribati; 5 Clinical Research Department, London School of Hygiene & Tropical Medicine, London, United Kingdom; 6 Kiritimati Hospital, London, Kiritimati Island, Kiribati; 7 International Trachoma Initiative, Task Force for Global Health, Decatur, Georgia, United States of America; 8 MRC Tropical Epidemiology Group, Infectious Disease Epidemiology Department, London School of Hygiene & Tropical Medicine, London, United Kingdom; 9 Division of Parasitic Diseases and Malaria, Centers for Disease Control and Prevention, Atlanta, Georgia, United States of America; 10 Department of Control of Neglected Tropical Diseases, World Health Organization, Geneva, Switzerland; RTI International, UNITED STATES

## Abstract

**Objective:**

In some Pacific Island countries, such as Solomon Islands and Fiji, active trachoma is common, but ocular *Chlamydia trachomatis* (*Ct*) infection and trachomatous trichiasis (TT) are rare. On Tarawa, the most populous Kiribati island, both the active trachoma sign “trachomatous inflammation—follicular” (TF) and TT are present at prevalences warranting intervention. We sought to estimate prevalences of TF, TT, ocular *Ct* infection, and anti-*Ct* antibodies on Kiritimati Island, Kiribati, to assess local relationships between these parameters, and to help determine the need for interventions against trachoma on Kiribati islands other than Tarawa.

**Methods:**

As part of the Global Trachoma Mapping Project (GTMP), on Kiritimati, we examined 406 children aged 1–9 years for active trachoma. We collected conjunctival swabs (for droplet digital PCR against *Ct* plasmid targets) from 1–9-year-olds with active trachoma, and a systematic selection of 1–9-year-olds without active trachoma. We collected dried blood spots (for anti-Pgp3 ELISA) from all 1–9-year-old children. We also examined 416 adults aged ≥15 years for TT. Prevalence of TF and TT was adjusted for age (TF) or age and gender (TT) in five-year age bands.

**Results:**

The age-adjusted prevalence of TF in 1–9-year-olds was 28% (95% confidence interval [CI]: 24–35). The age- and gender-adjusted prevalence of TT in those aged ≥15 years was 0.2% (95% CI: 0.1–0.3%). Twenty-six (13.5%) of 193 swabs from children without active trachoma, and 58 (49.2%) of 118 swabs from children with active trachoma were positive for *Ct* DNA. Two hundred and ten (53%) of 397 children had anti-Pgp3 antibodies. Both infection (p<0.0001) and seropositivity (p<0.0001) were strongly associated with active trachoma. In 1–9-year-olds, the prevalence of anti-Pgp3 antibodies rose steeply with age.

**Conclusion:**

Trachoma presents a public health problem on Kiritimati, where the high prevalence of ocular *Ct* infection and rapid increase in seropositivity with age suggest intense *Ct* transmission amongst young children. Interventions are required here to prevent future blindness.

## Author summary

Ocular infection with *Chlamydia trachomatis* causes trachoma. It is the leading infectious cause of blindness, and the target of an international campaign for elimination as a public health problem. Trachoma is endemic to Tarawa, the most populated island of Kiribati, housing approximately half the national population. However, the country has 20 inhabited islands and there were no previous trachoma prevalence data from Kiribati outside Tarawa. We set out to determine the prevalence of trachoma in the second most populated island, Kiritimati, located over 3000 km from Tarawa. In some other Pacific Island countries, ocular *C*. *trachomatis* infection is much less prevalent than the clinical signs that are used to guide interventions; we therefore looked for PCR-based evidence of current infection and antibodies to chlamydial proteins, in addition to recording clinical signs of trachoma. Our results indicate that trachoma and ocular *C*. *trachomatis* infection are prevalent on Kiritimati, and suggest that interventions are required here. The combined application of antibody, nucleic acid and clinical tools in an intervention-naïve population provides insight into their inter-relationships and the data are, therefore, of considerable interest to elimination programmes within and beyond the Pacific.

## Introduction

Trachoma is the leading infectious cause of blindness and the subject of an international campaign for elimination as a public health problem [1,2]. The strategy for elimination uses the acronym “SAFE”: surgery for advanced disease; and antibiotics, facial cleanliness and environmental improvement to clear infection and reduce transmission of *Chlamydia trachomatis* (*Ct*), the causative organism.

Australia [3] and several Pacific Island nations [4–6] have long been known to be trachoma-endemic. The first systematic investigation of trachoma in the Pacific was conducted in 2007, when a series of Trachoma Rapid Assessments was undertaken involving examination of 3102 children aged 1–9 years and 903 adults aged ≥40 years in 67 high-risk communities of Kiribati, Nauru, Vanuatu, Solomon Islands and Fiji [7].

In Kiribati, the 2007 Trachoma Rapid Assessments included 15 sites in Betio and Buota on Tarawa Atoll, in the far west of the country. A total of 655 children aged 1–9 years and 160 adults aged ≥40 years were examined; more than one-third of those children had trachomatous inflammation—follicular (TF), and nearly two-thirds of the adults had trachomatous conjunctival scarring (TS) [8]. One case of trachomatous trichiasis (TT) was identified, and it was reported that 10 TT surgeries had been performed locally (in a population of about 100,000 people) [9] over a twelve-month period. Trachoma Rapid Assessment data are deliberately biased to increase the likelihood that trachoma will be identified where it is endemic and therefore do not provide the prevalence estimates necessary to facilitate programme planning; these results provided justification for undertaking a formal prevalence survey.

**Fig 1:**
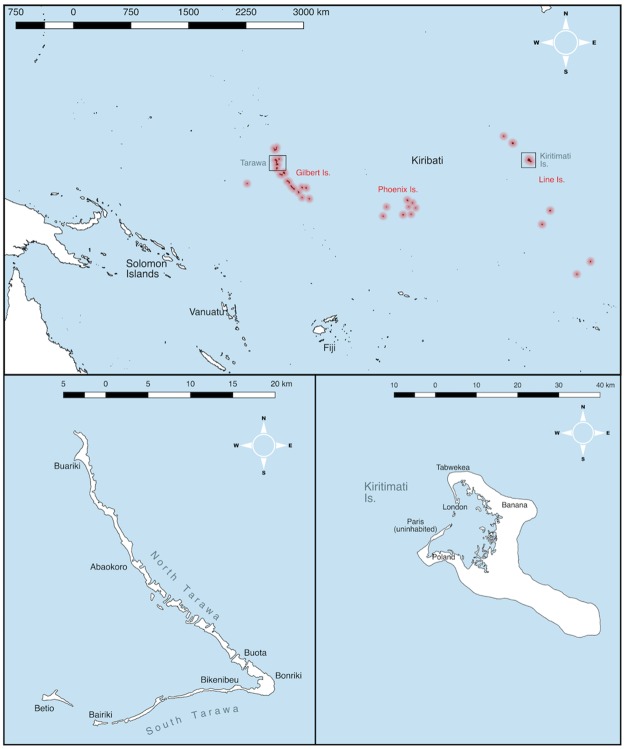
Map of Kiribati, with zoomed maps of Tarawa (lower left panel) and Kiritimati (lower right panel). Produced in Miller projection using QGIS 2.16. Shapefiles from gadm.org.

In 2012, therefore, a population-based prevalence survey was undertaken in Kiribati’s capital, South Tarawa, and the adjacent township of Betio (Fig 1). It estimated the unadjusted TF prevalence in children aged 1–9 years to be 21% and the unadjusted TT prevalence in adults aged ≥15 years to be 1.5% [10]. Because, in 2010, the combined population of South Tarawa and Betio was 49% of the national population [9], this survey could have been taken as evidence justifying implementation of the A, F and E components of the SAFE strategy [11] throughout Kiribati: even if there was no TF elsewhere in the country, the national TF prevalence would still have been greater than the 10% threshold above which WHO recommends A, F and E for at least three years before a repeat survey [12]. However, Kiribati is made up of 33 atolls and islands dispersed over 3.5 million square kilometers of ocean—approximately half the size of Australia—and the expense of rolling out programme-level interventions in a nation of many tiny islands dispersed over such an area would be considerable. In addition, recent evidence suggests that the epidemiology of trachoma in Vanuatu, Fiji and the Solomon Islands is different to that in much of trachoma-endemic Africa, with a relative paucity of TT [13] and low prevalences of ocular *Ct* infection for the observed prevalences of TF [14–17]. Whether or not TT and ocular *Ct* infection were present elsewhere in Kiribati was therefore a question of both public health and scientific significance.

Situated in the far east of Kiribati, Kiritimati Island of the northern Line Islands (Fig 1) has the greatest land area (388 km^2^) of any coral atoll in the world and comprises over 70% of the country’s total land area. It is the second-most highly populated island of Kiribati after Tarawa [9], with an estimated population in 2010 of 5586 people. We sought to estimate the prevalences of trachoma, ocular *Ct* infection, and anti-*Ct* antibodies on Kiritimati Island, Kiribati, in part to assess the relationships between these indices locally, and in part to guide planning processes for a national trachoma elimination programme.

## Methods

### Ethics statement

Ethics approval was received from the Research Ethics Committee of the London School of Hygiene & Tropical Medicine (reference numbers 6319, 8355 and 10136) and the Kiribati Ministry of Health and Medical Services (08/11/2015). Verbal consent to involve each village was obtained from its leaders. Written informed consent was obtained from all individuals examined, or a parent or guardian if the participant was aged <15 years.

### Study design

Children aged 1–9 years are the standard indicator group for estimating the prevalence of active trachoma [12]. We wished to have 95% confidence of estimating an expected TF prevalence in 1–9-year-olds of 20% with absolute precision of ±5%. Using the single population proportion for precision formula and correcting for a small, finite population (because the estimated number of 1–9-year-olds resident on Kiritimati was 1222), and using a design effect of 2.65, our sample size estimate was 426 1–9-year-olds [9,18,19]. Although we determined the sample size as a number of children, all individuals aged ≥1 year resident in selected clusters and who gave consent to be examined were included in the survey [19]. All four inhabited villages (the smallest administrative unit) on Kiritimati Island were visited (Fig 1), with the number of households to be invited to participate in each village proportional to that village’s relative size: 62 households from London, 106 households from Tabwekea, 42 households from Banana, and 11 households from Poland (Fig 1). Maps of each village were prepared by hand and compact segment sampling used, with clusters of households selected by random draw. In addition to household visits, data were collected at Presbyterian and Catholic *maneabas* (large open community halls where families gather and often stay for months) within or adjacent to the selected clusters.

### Clinical examination

Graders were trained and certified according to the standardized training protocols of the GTMP [19]. Trachoma grading was undertaken according to the WHO simplified grading scheme [8], using 2.5× binocular magnifying loupes and sunlight illumination. Eyes with trichiasis were considered to have TT if and only if they also had TS. (The presence or absence of TS was not recorded in the absence of trichiasis.) Graders cleaned their hands with alcohol-based hand gel after each examination.

Participants identified as having active trachoma (TF and/or trachomatous inflammation—intense [TI] in one or both eyes) were provided with two tubes of 1% tetracycline eye ointment, and they or their parents or guardian were instructed on how to apply it. Participants with trichiasis were referred for management by a trained ophthalmologist.

### Specimen collection and handling

When a 1–9-year-old child was found to have TF and/or TI, a conjunctival swab was taken by the examiner. In addition, a conjunctival swab was taken from a systematic two-thirds sample of 1–9-year-old children without these signs, with the creation of this sub-sampling approach necessitated by the discovery that a box of sterile swabs had been misplaced in transit to the island, prior to fieldwork—this meant that we would not be able to swab all the children that we intended to examine. Systematic sampling was achieved by selecting the first two of every three children without active trachoma, in order of presentation. Individuals older than 9 years were not swabbed, regardless of whether or not they had active trachoma. In subjects selected for swabbing, a single sterile polyester-coated swab (Puritan Medical Products, Guilford, USA) was passed four times across the upper tarsal conjunctiva of the more inflamed eye, rotating the swab 90° after each pass. Using a technique that minimised the likelihood of the retained part of the swab shaft or swab-head touching anything other than the subject’s conjunctiva and the collection tube, swabs were placed in polypropylene tubes and refrigerated at the end of each day; ice was not available locally. Following grading and (if indicated) swab collection, examiners collected fingerpick blood onto filter paper (Cell Labs Pty, Sydney, Australia) from all 1–9-year-old children assessed. Filter papers were air-dried overnight in an air-conditioned room, then packed in individual resealable plastic bags; up to 100 of those primary plastic bags were enclosed in large secondary resealable plastic bags containing dessicant sachets for refrigeration and transport. Swabs and dried blood spots were shipped at ambient temperature to the London School of Hygiene & Tropical Medicine for droplet digital PCR (ddPCR) and anti-*Ct* antibody testing, respectively. Handling and processing was identical for all samples, being undertaken masked to examination results.

### ddPCR for ocular *Chlamydia trachomatis* infection

Genomic DNA was extracted from swabs using the QIAamp DNA mini kit (Qiagen, Manchester, UK). Proprietary lysis buffer and proteinase K were added directly to the specimen collection tube and incubated for 1 hour at 56°C to lyse the specimen. The DNA was then bound to a silica spin column, washed, and eluted into 100μL Tris-Cl EDTA. One 8μL aliquot was tested for conserved regions of *Ct* plasmid open reading frame 2 (diagnostic target) and human 30kDa ribosomal RNA subunit (RPP30; endogenous control target) using an in-house ddPCR, described elsewhere [16,20].

### Serological testing for anti-Pgp3 antibodies

An area of each filter paper calibrated to hold 10μL of blood was tested using an ELISA for anti-Pgp3 antibodies [14,21]. Serum was eluted from dried blood spots overnight at 4°C into phosphate-buffered saline (PBS) supplemented with 0.3% volume/volume Tween 20 (PBST) and 5% milk powder, for a final serum dilution of 1:50. Immulon^®^ 2HB plates were coated with GST-tagged Pgp3 overnight at 4°C. The next day, plates were washed and then blocked with PBST for 1h at 4–10°C. After removal of blocking buffer, 50μL of the serum elution was added to each well and plates incubated at room temperature for 2h on an orbital shaker. After removing unbound antibody with PBST, 50μL of rabbit anti-human IgG (Southern Biotech, Brimingham, USA) diluted 1:32,000 in PBST was added to each well, and plates were incubated for 1h at room temperature on an orbital shaker. After washing with PBST, plates were incubated in the dark at room temperature for 10 minutes with 50μL per well TMB (KPL, Gaithersburg, USA). The reaction was stopped with 50μL 1N H_2_SO_4_ and the absorbance read at 450nm on a Spectramax M3 plate reader (Molecular Devices, Wokingham UK). Readings were corrected for background by subtracting the average absorbance of three blank wells containing no serum, using Softmax Pro5 software (Molecular Devices). Serial dilutions of high-titre serum in low-titre serum were run on each plate, and all sample ODs were normalized against that of a middle dilution of the high-titre serum. A finite mixture model [21–23] was used to estimate a negative population in the resulting normalised optical density (NOD) values, and specimens were considered anti-Pgp3 positive if their NOD was more than three standard deviations above the mean of the presumed-negative population (a NOD threshold of 0.245).

### Data recording and analysis

Data entry and real-time server upload was via a bespoke android-based Open Data Kit data capture system, developed as part of the GTMP [19]. Participant data were encrypted and stored in a secure server with only the study investigators having access. TF prevalence data were adjusted for the age of those examined, in five-year age bands. TT prevalence data were adjusted for age and gender of those examined, in five-year age bands. The most recent census data [9] were used as the reference dataset for the purposes of undertaking these adjustments. Results are presented in accordance with STrengthening the Reporting of OBservational studies in Epidemiology (STROBE) guidelines (see S1 Checklist) [24].

## Results

### Clinical findings

Fieldwork was conducted in November 2015. This coincided with the tenth anniversary of the arrival of Christianity to Kiritimati; as a consequence of the associated celebrations, a majority of families had decamped to local *maneabas*. We examined 406 of the 412 children aged 1–9 years identified as being resident in selected segments; the other six children were absent at the time of the field teams’ visits and could not be located by their parents or guardians. Of those examined, 123 children had TF and 16 had TI; all children with TI also had TF. The age-adjusted prevalence of TF in children was 28% (95% CI 24–35). Of 417 adults aged ≥15 years enumerated, 416 were examined, with one person refusing to participate. A total of 8 adults were identified to have TT. The age- and gender-adjusted prevalence of TT in adults was 0.2% (95% CI 0.09–0.33%). One person had bilateral TT.

### Ocular *Chlamydia trachomatis* infection

Swabs were available from 311/406 (77%) children aged 1–9 years: 118 children with TF and/or TI, and 193 systematically selected children without either of these signs. Infection was significantly associated with signs of active trachoma (Table 1; Pearson’s Χ^2^ p<0.0001). Extrapolating the infection prevalence in swabbed children who had neither TF nor TI (13.5%) to all such children (including the 90 who were not swabbed), we expect that 38 of the 283 children who had neither TF nor TI were infected; extrapolating the infection prevalence in swabbed children with TF and/or TI (49.2%) to all such children (including the 5 who were not swabbed), we expect that 60 of the children with TF and/or TI were infected. This would give an estimated overall prevalence of infection in examined 1–9-year-olds of 24%.

**Table 1:** Infection with *Chlamydia trachomatis* in 1–9-year-old children, with and without active trachoma in either eye, Kiritimati Island, Kiribati, November 2015. TF = trachomatous inflammation—follicular; TI = trachomatous inflammation—intense; ddPCR = droplet digital polymerase chain reaction.

		ddPCR		
		Negative (%)	Positive (%)	Total (%)
**Examination findings**	**No TF or TI**	167 (86.5)	26 (13.5)	193 (62.1)
	**TF and/or TI**	60 (50.8)	58 (49.2)	118 (37.9)
	**Total**	227 (73.0)	84 (27.0)	311 (100.0)

**Fig 2:**
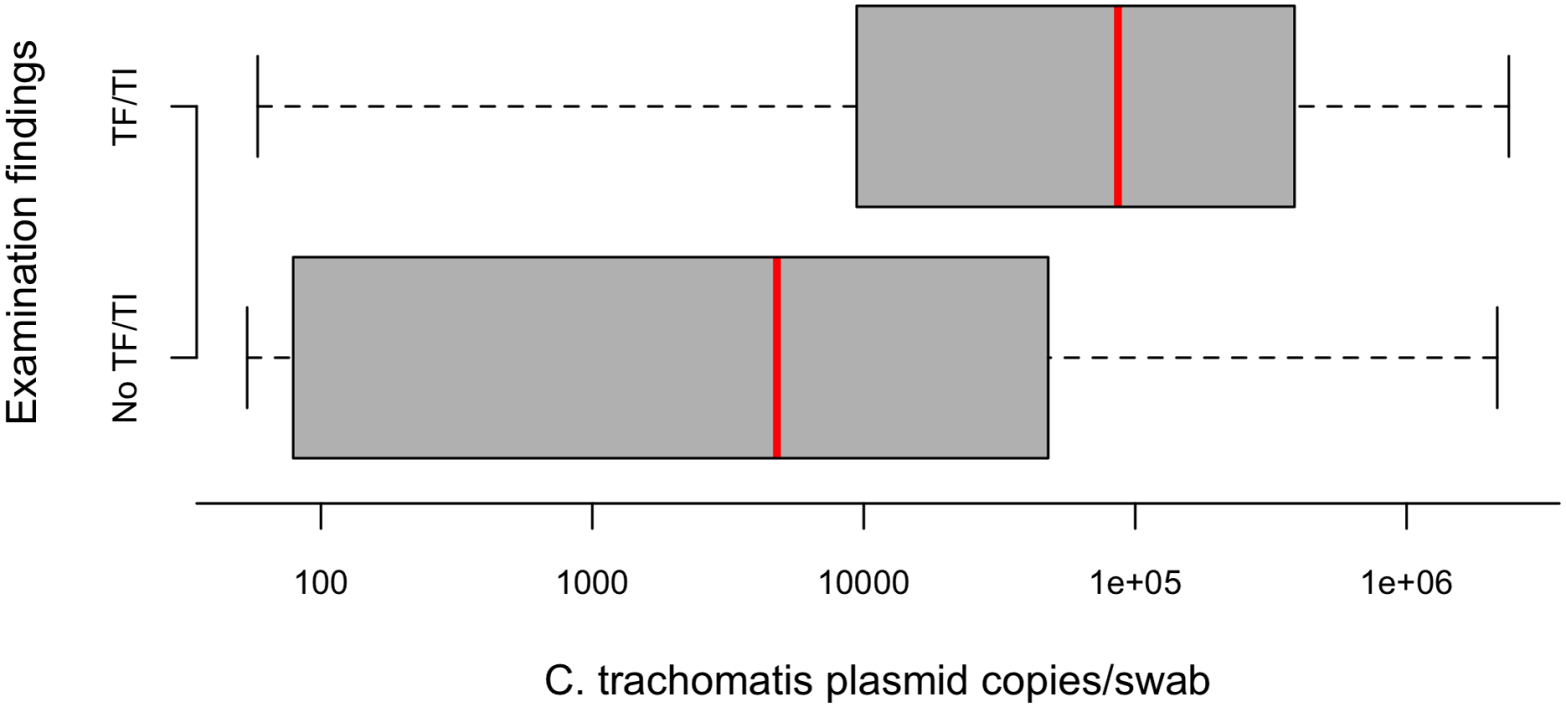
Load of *Chlamydia trachomatis* infection in 1–9-year-old children with and without active trachoma in either eye, Kiritimati Island, Kiribati, November 2015. Red lines represent the median value, grey boxes represent inter-quartile ranges and whiskers represent the minima and maxima. TF = trachomatous inflammation—follicular; TI = trachomatous inflammation—intense.

A median of 17,330 RPP30 copies were found per swab, equivalent to approximately 8,500 host cells per swab. Of PCR-positive swabs (n = 84), the median *Ct* load in children with TF and/or TI in either eye was significantly higher than in those with neither TF nor TI (86,450 vs 6,530 plasmid copies/swab, logistic regression p = 0.0057; Fig 2).

### Anti-Pgp3 antibodies

**Fig 3:**
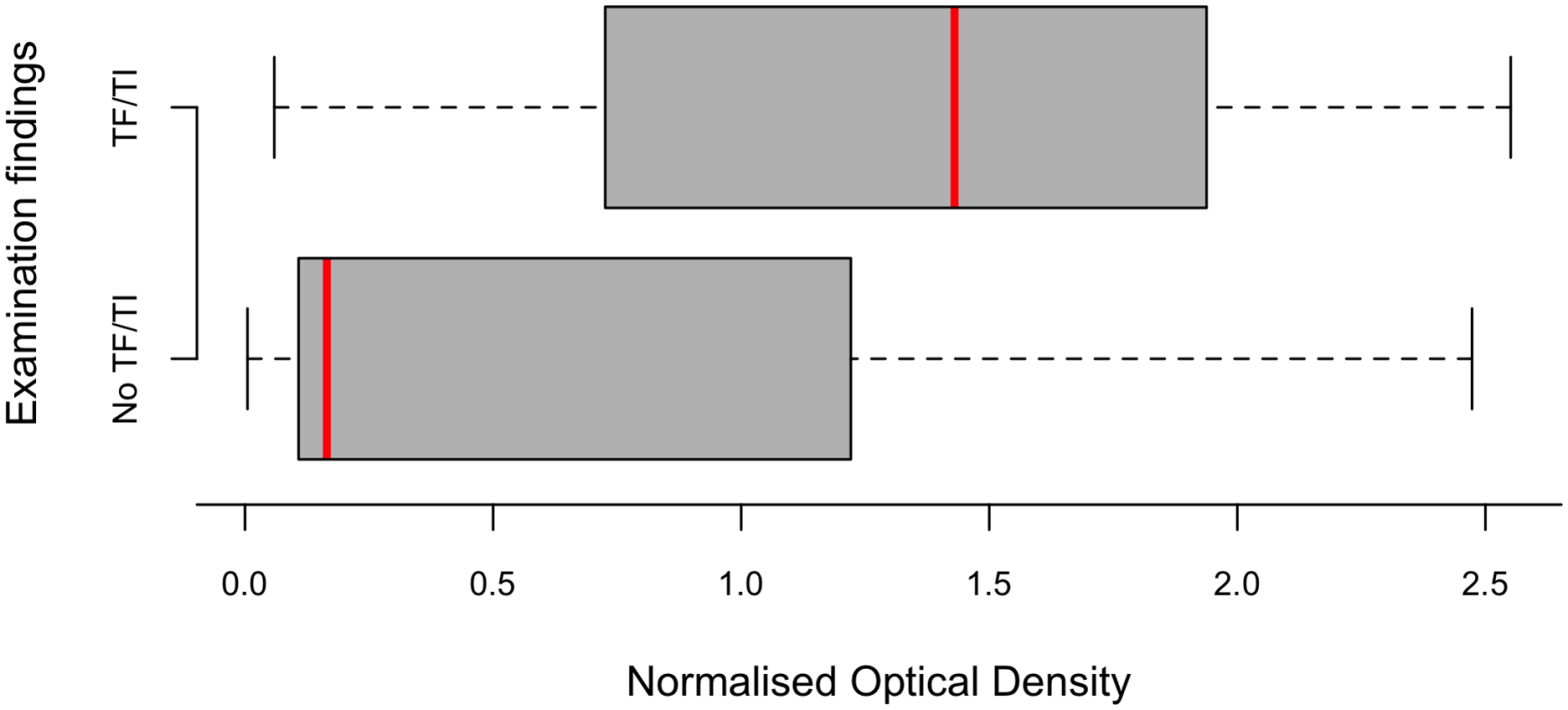
Normalized optical density (in an ELISA for anti-Pgp3 antibody) in sera from 1–9-year-old children with and without active trachoma in either eye, Kiritimati Island, Kiribati, November 2015. Red lines represent the median value, grey boxes represent inter-quartile range and whiskers represent the minima and maxima.

**Fig 4:**
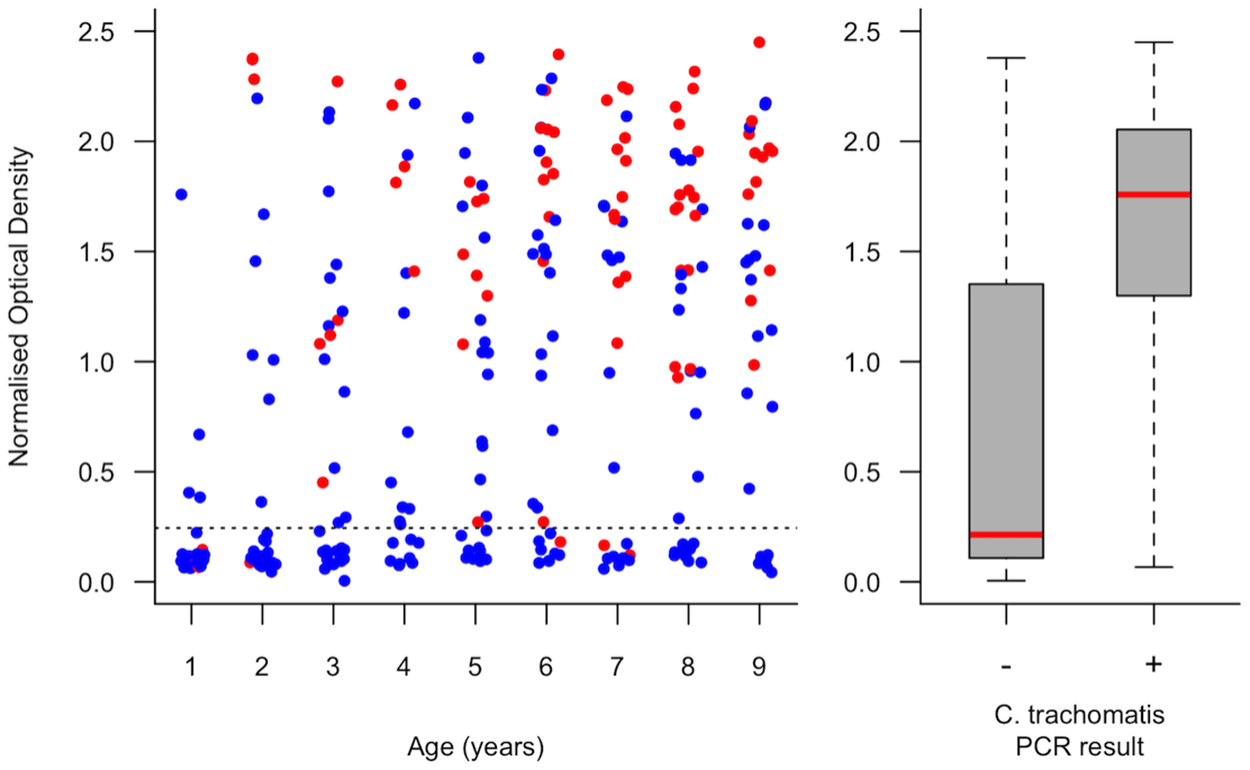
Normalized optical density (in an ELISA for anti-Pgp3 antibody) in sera from 1–9-year-old children who were positive (red spots in panel (a)) and negative (blue spots in panel (a)) for conjunctival *Chlamydia trachomatis* infection by droplet digital PCR, Kiritimati Island, Kiribati, November 2015. In panel (a), the threshold for anti-Pgp3 seropositivity, derived using a finite mixture model, is indicated (dotted line), and within each one-year age band, spots have been moved horizontally in order to separate data points that would otherwise have been superimposed. In panel (b), red lines represent the median values, grey boxes represent inter-quartile ranges, whiskers represent the minima and maxima.

**Fig 5:**
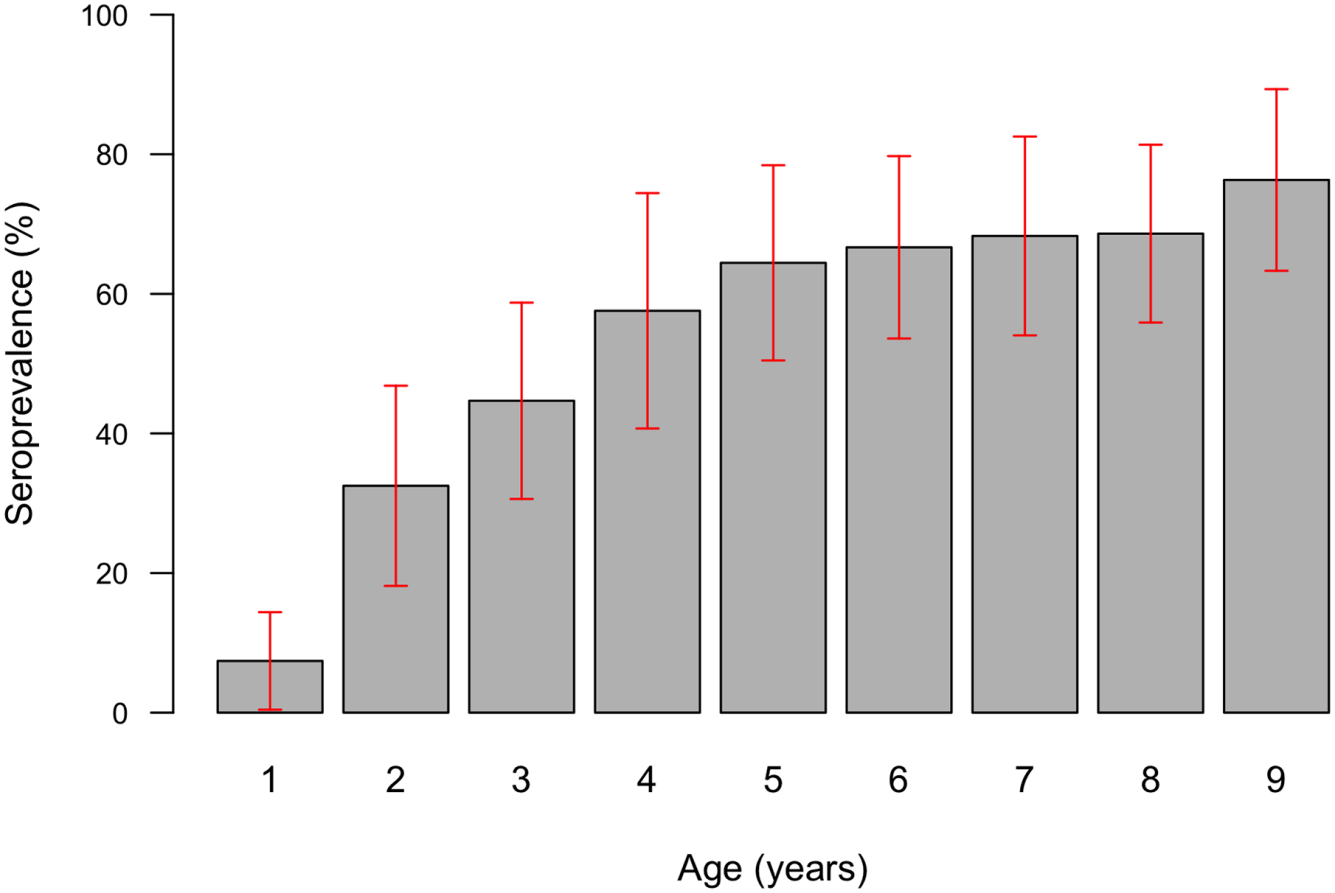
Age-specific prevalence of anti-Pgp3 antibodies in 1–9-year-old children, Kiritimati Island, Kiribati, November 2015. Grey columns indicate the seroprevalence estimate for each one-year age band; red lines indicate 95% confidence intervals around those estimates.

Blood spots were analysed from 397 children aged 1–9 years; 8 of the remaining 9 examined children did not assent to fingerprick blood collection, and 1 child was not bled because his parents reported a bleeding diathesis. Seropositivity was significantly associated with active trachoma (TF and/or TI in either eye) (Χ^2^ p<0.00001; Table 2). Reactivity to Pgp3 was higher in children with active trachoma than in those with neither TF nor TI (Fig 3), and significantly higher in those with conjunctival *Ct* infection than in those without (logistic regression p<0.0001, Fig 4). 90.7% of PCR-positive children were seropositive. The age-specific seroprevalence was low in those aged 1 year (7%), increased rapidly between the ages of 1 and 5 years, and was 76% in those aged 9 years (Fig 5). This increase in seropositivity with age was highly significant (logistic regression p = 0.0007). There was also a significant increase in NOD with age (logistic regression p<0.0001).

**Table 2:** Reactivity to Pgp3 in 1–9-year-old children with and without active trachoma in either eye, Kiritimati Island, Kiribati, November 2015. TF = trachomatous inflammation—follicular; TI = trachomatous inflammation—intense.

		ELISA		
		Negative (%)	Positive (%)	Total (%)
**Examination findings**	**No TF or TI**	164 (59.0)	114 (41.0)	278 (70.0)
	**TF and/or TI**	23 (19.3)	96 (80.7)	119 (30.0)
	**Total**	187 (47.1)	210 (52.9)	397 (100.0)

## Discussion

We have estimated the prevalence of trachoma and current and cumulative infection with *C*. *trachomatis* on Kiritimati Island. Our GTMP-based approach to sampling via household selection [19] was challenged by the extensive community bonds of the Kiritimati population, which mean that nuclear families tend not to maintain exclusive residence in a single family dwelling. Prolonged spells of communal living in *maneabas* and individual mobility between households make the question, “Where do you live?” difficult for residents to answer. Rather than undertaking a complete census of the island’s population and selecting individuals by simple random sampling, we opted for compact segment sampling, including the geographically adjacent *maneaba* or *maneabas*. This was an imperfect solution, in that it will have somewhat biased survey recruitment by including individuals not normally resident within the land area of the selected segment. We did not keep a record of whether individual participants were examined in their home or in a *maneaba*, entering data on all subjects as though they were residents of the same household. A consequence of this was that we were unable to adjust for clustering in our analyses, which may result in our prevalence estimates being less precise than the confidence intervals that we present here. A further limitation was the lack of access to ice to keep samples cold in the field, where temperatures peaked at >30°C each day. We carried samples in insulated boxes, then refrigerated them at the end of the day’s work and until they were transported to Manila, Philippines, for repackaging and shipment to London. DNA degradation occurs more rapidly at higher temperatures, and it is therefore possible that our estimates of *Ct* PCR positivity and DNA loads are underestimates, however our own [15] and others’ [25] data suggest this degradation results in (qualitative) diagnostic failure only in very low load samples, which are presumably from individuals least likely to pass infection on to contacts [26]. Loss of antibody following storage for several hours at temperatures >30°C is minimal [27]. Finally, although we took great care to avoid carry-over contamination of swabbed material from one subject to the next, we cannot say with certainty that this did not occur.

Notwithstanding those limitations, our data suggest that trachoma is a public health problem on Kiritimati Island. The prevalence of TF in 1–9-year-olds observed here (28%) is at a level at which WHO recommends implementation of the A, F and E components of the SAFE strategy for at least three years before re-survey [12]. The Kiritimati TF prevalence that we observed is broadly comparable to that (21%) estimated for South Tarawa and Betio of Kiribati in 2012. Whether these two survey findings, taken together, should be extrapolated as the basis for a decision to deliver population-based interventions, including mass drug administration of azithromycin [28], across the country’s 18 other inhabited islands, is a question that will require considerable thought. We submit that such an approach is now justified.

The prevalence of TT that we observed in adults—0.21%, though two significant digits may be more than is justified given that there were only 8 cases—is just over the TT threshold of 0.2% specified by WHO as part of the definition for trachoma’s elimination as a public health problem [29]. Ongoing provision of TT surgical services on Kiritimati is likely to be necessary, but because of the relatively small total population, the number of people needing such services is likely to be small. The finding that there is a burden of TT here is again consistent with the situation in South Tarawa and Betio, but contrasts with that in several other Pacific Island countries in which trachoma mapping has been undertaken to date, where TT is rare to absent in adults despite moderate prevalences of TF in children.

We found strong evidence for both current *Ct* infection (demonstrated by PCR-positive conjunctival swabs) and widespread prior exposure to *Ct* infection (demonstrated by antibodies to Pgp3) in children on Kiritimati. Both the presence and load of infection was closely associated with active trachoma in this population, as is typical of trachoma-endemic environments in sub-Saharan Africa [30]. In contrast, in trachoma-endemic communities of the Solomon Islands and Fiji, the prevalence of ocular *Ct* infection has recently been shown to be very low; it has been hypothesized that the latter finding may account, in whole or in part, for the scarcity of trichiasis in those environments [13,15,16].

TI is associated with chronic, repeated conjunctival *Ct* infection and incident TS [31–33], the precursor to TT. Of the 406 children aged 1–9 years that we examined, 16 (4%) had TI, compared to 2 (0.2%) of 1135 children aged 1–9 years examined in the Solomon Islands population in which the prevalence of *Ct* was only 1.3% [16]. The high prevalence of antibody positivity found in the children bled on Kiritimati indicates a high prevalence of previous exposure to *Ct* infection, and the steep increase in seroprevalence we observed between those aged 1 and 5 years suggests intense transmission is occurring in that age group [34,35]. These observations all point to a significant force of infection present in the Kiritimati population, making it straightforward to understand the occurrence of TT in adults, and underscoring the need for implementation of interventions to reduce the prevalence and transmission of infection here. As elimination activities progress, re-measuring the prevalence of conjunctival *Ct* infection and prevalence of anti-Pgp3 antibodies in young children will be instructive, with those longitudinal data likely to help us to further understand the potential utility of these tools for monitoring programmes [36].

Our finding that a substantial proportion of 1–9-year-olds with neither TF nor TI were *Ct*-positive (13%, Table 1) and anti-Pgp3 antibody-positive (41%, Table 2) is not new [37–40], and has been the source of prolonged discussion over how best to evaluate the potential programmatic use of laboratory tests for *Ct* exposure [41–44]. It is certainly true that the WHO simplified trachoma grading scheme is relatively insensitive, ignoring degrees of conjunctival inflammation less marked than five moderately-sized central follicles or pronounced thickening obscuring at least half of the normal deep tarsal blood vessels [8]. Some children who had neither TF nor TI undoubtedly had lesser degrees of inflammation than this. More sensitive, more detailed trachoma grading systems exist [45], but the simplified system was specifically developed because those systems were felt to be too complex for use by non-specialist personnel working at community level [8], and is the current standard against which any proposed new method for evaluating the success of interventions against trachoma should be assessed.

The differences observed in the epidemiology of trachoma here with that in neighbouring countries could be due to a number of different factors. Kiribati’s population is predominantly Micronesian, whereas the island nations to the southwest are dominated by peoples of Melanesian descent. The environment is also different: despite the abundant vegetation, fresh water availability is poor throughout Kiribati because infrastructure is inadequate, the evaporation rate is high, droughts are frequent, and the soil is mostly loose and drains quickly [46]. Such conditions are associated with greater risk of trachoma elsewhere: in a national survey of Nigeria, for example, villages with hotter, drier climates have greater risk of TT than cooler, wetter villages [47]. Because the preponderance of people enrolled in this study were examined in *maneabas* rather than in their own homes, collecting data on household-level water and sanitation risk factors for trachoma was not undertaken [48–50], but doing so in future data collection exercises here, with appropriate adjustments in the unit of data collection (to account for communal living), would be of interest.

Our data indicate a need for interventions against trachoma in this population, and will serve as a baseline for ongoing comparative studies of the epidemiology and pathogenesis of trachoma in the Pacific Islands.

## Supporting information

S1 Table Raw data from this study in accordance with journal data availability guidelines. (CSV)

S1 Checklist STROBE checklist. (DOC)
